# Comparative analysis between slow freezing and ultra-rapid freezing for human sperm cryopreservation

**DOI:** 10.5935/1518-0557.20180060

**Published:** 2018

**Authors:** Natalí S. Riva, Claudio Ruhlmann, Rocío S. Iaizzo, Carla A Marcial López, Alejandro Gustavo Martínez

**Affiliations:** 1 Fertilidad San Isidro, Buenos Aires, Argentina

**Keywords:** human spermatozoa cryopreservation, slow freezing, non-permeable cryoprotectant, ultra-rapid freezing, vitrification, DNA fragmentation

## Abstract

**Objective:**

Cryopreservation of human spermatozoa is fundamental in assisted reproductive technology. At present, slow freezing techniques are widely used for sperm cryopreservation. Recently, sperm vitrification has been proposed as an alternative to slow freezing. This study aimed to compare the efficiency of slow versus ultra-rapid freezing after thawing and to determine the level of DNA fragmentation in post-thaw normal human semen samples processed through each of the cryopreservation techniques.

**Methods:**

Ultra-rapid freezing is a method that only differs from conventional ultra-rapid freezing in the use of sucrose as a cryoprotectant. In experiment 1, 24 semen samples were used to compare sperm recovery rates after slow and ultra-rapid sperm freezing. In experiment 2, 18 semen samples were used to compare post-thaw sperm DNA fragmentation levels after each of the cryopreservation techniques.

**Results:**

In experiment 1, no significant differences were observed in sperm concentration recovery rates, although slow freezing showed a lower progressive motility rate than ultra-rapid freezing (16.6±7.4% vs. 34.7±10.2%), and higher non-progressive and immotile sperm counts (9.0±4.0% vs. 7.6±2.8%; and 74.4±10.1% vs. 57.8±10.3%, respectively). In experiment 2, sperm DNA fragmentation after thawing was significantly higher in slow freezing than in fresh post gradient processing and ultra-rapid freezing samples (47.3±13.4% vs. 9.1±3.7% vs. 14.6±4.6%, respectively).

**Conclusion:**

Sperm ultra-rapid freezing may be an alternative to slow freezing with better recovery results and less apparent DNA damage.

## INTRODUCTION

Cryopreservation of human spermatozoa is a fundamental resource in assisted reproductive technology (ART) that allows the optimization of infertility treatment and male fertility preservation therapy prior to chemotherapy, radiotherapy or testicular radical surgery (Sanger *et al*., 1992). At present, slow freezing techniques have been widely used in sperm cryopreservation, allowing the storage of large sample volumes with acceptable results for sperm vitality and motility after thawing (Fuller *et al*., 2004; Donnez & Kim, 2011). Sperm vitrification has been proposed as an alternative to slow freezing (Isachenko *et al*., 2003).

Vitrification has proven its effectiveness from oocytes to embryos. These have been possible due to an acceleration of the cryopreservation process by minimizing the volumes of the solution with the development of different vitrification devices and the optimization of the cryoprotectant combination. A more efficient induction of the vitreous phase became viable, thus minimizing the toxic effect of cryoprotectants and leading to a marked improvement of the results. Consequently, vitrification is accepted today as the standard procedure for embryo and oocyte cryopreservation. The method described by Isachenko *et al*. (2003; (2004; 2008) and Isachenko *et al*. (2004; 2005) for sperm vitrification only differs from the technique developed for oocytes and embryos in the use of sucrose as the only cryoprotectant.

Recently published clinical studies in ART reported correlations between sperm DNA damage, poor embryo quality after fertilization, recurrent implantation failure, miscarriage, and congenital defects in the offspring (Seli & Sakkas, 2005; Aitken & De Iuliis, 2007; Cohen-Bacrie *et al*., 2009; Lewis *et al*., 2013). Controversial data have been published on the direct impact of sperm cryopreservation techniques on sperm DNA integrity (Kopeika *et al*., 2015; Ortega Ferrusola *et al*., 2010).

The aim of this study was to compare the efficiency of slow freezing versus ultra-rapid freezing in sperm cryopreservation in terms of viable sperm recovery rates and DNA fragmentation levels after thawing.

## MATERIAL AND METHODS

### Experimental design

A total of 42 semen samples donated for investigation purposes were received by the andrology lab of our private infertility clinic between February and December 2015. Donors were aged 34±4.1 years (Range: 28-39). Informed consent was obtained from all participants.

The study was carried out in accordance with the principles of the Declaration of Helsinki and was approved by the Institutional Ethics Committee.

### Inclusion criteria:

- Semen volume ≥1.5 ml
- Sperm concentration ≥20 x 10^6^ spermatozoa/ml
- Sperm motility: Progressive motility [10] ≥25%

### Experiment 1: Comparative analysis of sperm recovery after slow freezing and ultra-rapid freezing

Twenty-four raw semen samples were used to evaluate sperm recovery after slow freezing and ultra-rapid freezing. After quantification of sperm concentration and motility, the fresh samples were divided into two equal volume fractions and cryopreserved by the two techniques, as described below. After one to seven days in liquid nitrogen, the samples were processed according to each respective protocol. Sperm concentrations and motility were then evaluated.

### Experiment 2: Comparative analysis of sperm DNA fragmentation after slow and ultra-rapid freezing.

A different set of 18 semen samples was used to compare sperm DNA damage induced by the slow and the ultra-rapid freezing procedures. Each fresh sample was split in three fractions. One of the fractions was processed fresh by gradient separation to analyze DNA fragmentation values prior to cryopreservation. The remaining two fractions were processed with either slow or ultra-rapid freezing techniques and stored in liquid nitrogen. Finally, all samples were thawed as described below, and sperm DNA fragmentation levels were measured using the TUNEL technique and compared with DNA fragmentation values prior to cryopreservation.

### Fresh semen sample evaluation

Five microliters of each fresh sample were placed in a Makler counting chamber (Sefi-Medical Instruments, Haifa, Israel) and observed with a conventional binocular microscope (Olympus CH2, Tokyo, Japan). As described in the WHO Laboratory Manual (WHO, 2010), concentration and motility were determined after counting 100 sperm. The analysis was performed independently by two of the authors for each of the samples.

### Slow freezing technique

Each complete semen sample was placed in a 15-ml centrifuge tube (Nunc International, Roskilde, Denmark), diluted 1:1 with TEST-yolk buffer (Irvine Scientific, Ca, USA) in a slow drop wise manner, gently mixing it to form a homogeneous solution. This solution was equally aliquoted into four cryogenic vials (Nunc International, Denmark), previously labeled with the individual ID of each sample. The cryogenic vials were cooled down to 4-8ºC for 60 min and then from -12ºC to -18ºC for 5 more min. They were then held for another 5 min in nitrogen vapor (-170ºC to −180ºC). Finally, the samples were plunged into liquid nitrogen (-196ºC) in a labeled aluminum straw for storage (Nallella *et al*., 2004; Royere *et al*., 1996).

For thawing, the straws were removed from the liquid nitrogen tanks and the cryogenic vials were placed on a hot plate at 37ºC until thawing was complete. Then, the contents were placed in a 15-ml centrifuge tube, diluted 1:1 with modified human tubal fluid solution (mHTF; Irvine Scientific, Santa Ana, Ca, USA) containing 3% synthetic serum substitute (SSS), (Irvine Scientific, USA) at 37ºC, while being gently mixed. The samples were centrifuged for 10 min at 300*g*. The supernatant was removed and the pellet was suspended in 1 ml of mHTF plus 3% SSS. The resulting sample was processed in a 50/90% Isolate Gradient (Irvine Scientific, USA) and was washed and diluted in 0.4 ml of mHTF plus 3% SSS, to reach a final volume of 0.5 ml.

### Sperm ultra-rapid freezing technique

**Figure 1:**
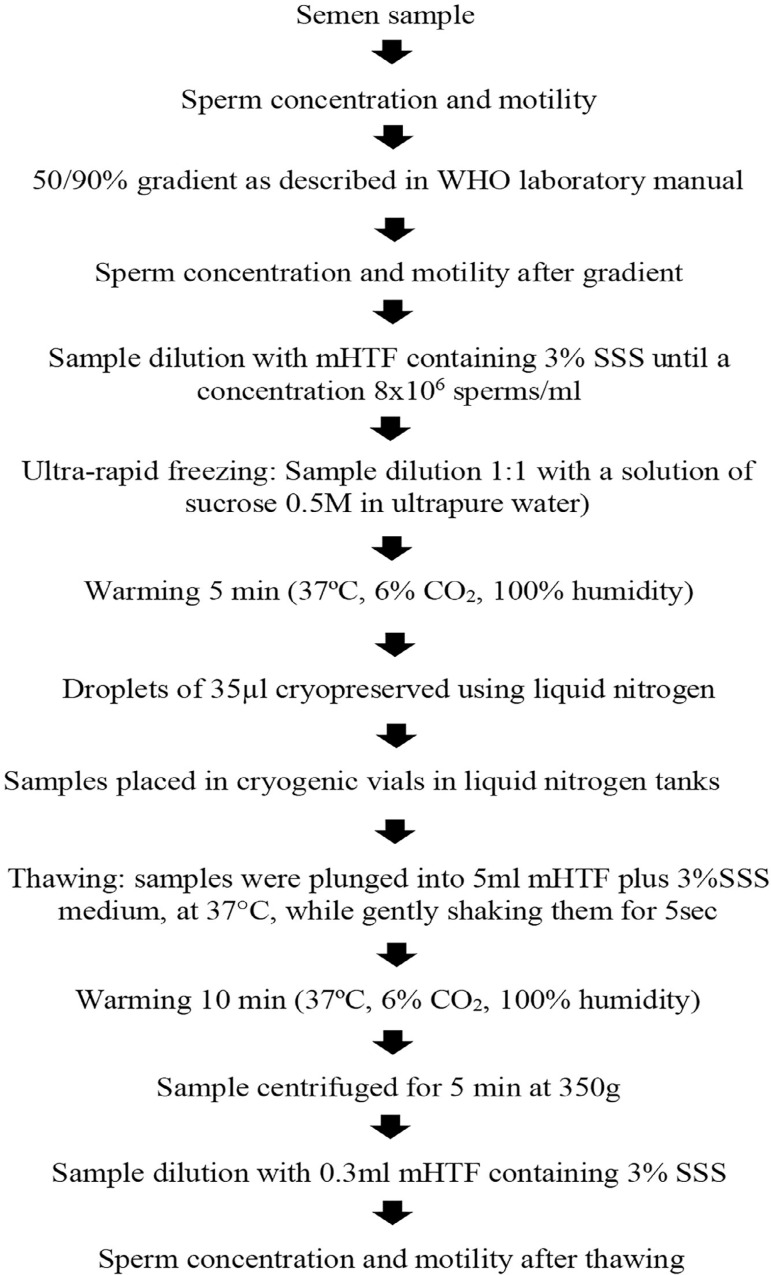
Ultra-rapid protocol.

A modification of the protocol described by Isachenko *et al*. (2005) was applied, as described below (Fig. 1). Sperm selection was performed using a 50/90% Isolate gradient as described in the WHO laboratory manual (WHO, 2010). The resulting solution was diluted 1:1 with 0.5M sucrose solution (Merck, Darmstadt, Germany) in mHTF containing 3% SSS. Then it was incubated at 37ºC for 5 more min, and a liquid nitrogen container with a strainer was prepared to receive the processed samples. Droplets of 35µl were cryopreserved, by placing a 0-200µl manual pipette at a 45º angle, 10 cm away from the nitrogen surface. One droplet was added every 5 secs, allowing it to solidify and sink to the bottom of the strainer. For storage, the samples were placed in cryogenic vials in liquid nitrogen tanks for at least 24h.

For thawing, the samples were plunged into 5ml mHTF plus 3%SSS medium at 37ºC, while gently shaken for 5 secs. The resulting suspension was incubated at 37ºC for 10min. Then it was centrifuged for 5min at 350 *g,* and finally added to 0.4ml of a mHTF + 3%SSS solution, to reach a final volume of 0.5ml.

### Sperm DNA fragmentation determinations

A TUNEL assay (Terminal deoxynucleotidyl transferase dUTP nick end labeling assay) was employed to determine sperm DNA fragmentation levels, as described by Rougier *et al.* (2013). Special glass slides (Teflon printed slides for TUNEL; EMS, Madison, WI, USA) were immersed for two hours in a solution of 0.01% poly-l-lysine in ultra-pure water (Merck, Darmstadt, Germany), and were then washed with ultra-pure water and left to dry at room temperature. The processed samples were fixed in 37% formaldehyde (Merck, Darmstadt, Germany) and stored at 4-8ºC until evaluation.

**Figure 2:**
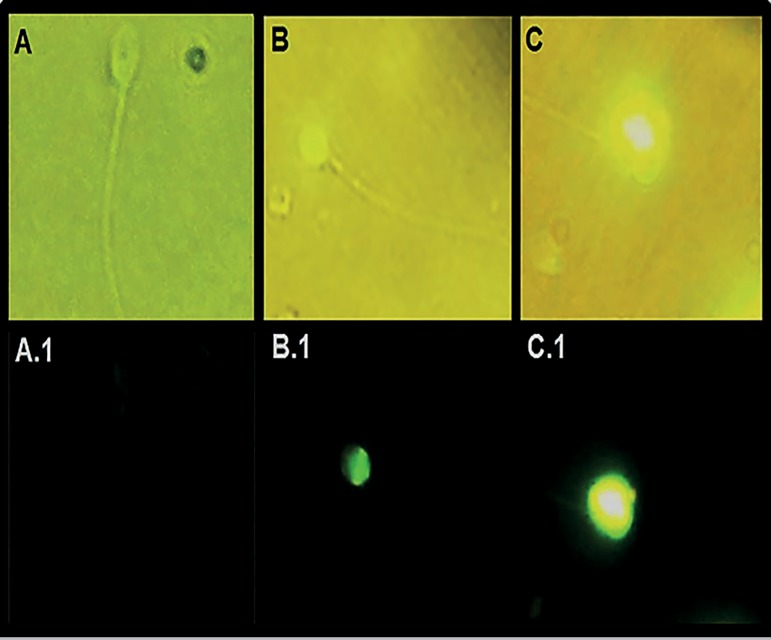
Spermatozoa with different TUNEL labeling levels (100X).**** **A**. TUNEL-negative spermatozoa with 0% fluorescence under white light. **A.1**. The same spermatozoa under UV light. **B**. Negative spermatozoa with <50% fluorescence under white light. **B.1**: The same spermatozoa under UV light. **C.** Positive spermatozoa with >50% fluorescence under white light. **C.1.** The same spermatozoa under UV light.

To evaluate DNA fragmentation, 30-µl aliquots of the samples were spread in duplicate on the slides, and were then incubated in humidified chambers for 24h at 4-8ºC. Next, the samples were rinsed three times over 5min, with 10µl of phosphate-buffered saline solution (PBS 1X, Irvine Scientific, Ca, USA). They were then placed in methanol (Merck, Darmstadt, Germany) for 90 seconds, and rinsed again three times with PBS 1X. The slides were placed in 10µl of PBS buffer solution added with 0.5% bovine serum albumin (Bovine Serum Albumin; Merck, Darmstadt, Germany) for 50min in a humidified chamber at 4-8ºC, and were then washed again three times with PBS 1X. The slides were treated with 4.5µl of label and 0.5µl of enzyme (In Situ Death Cell Detection Kits, Roche Diagnostics, Minneapolis, MN, USA) and placed in a humidified chamber for 1 hour in the dark. The slides were then rinsed 3 times over 5min with 10µl of PBS 1X, and dried at room temperature in the dark. Finally, 5µl of Vecta Shield antifade mounting medium (Vector Laboratories, Burlingame, CA, USA) was added to each slide, and they were covered with 24x50mm coverslips. Two operators examined the samples on a fluorescence microscope (Mikoba S320, Beijing, P.R. China) at 100X magnification under immersion oil. Apoptotic spermatozoa were counted from 200 cells. Spermatozoa with >50% fluorescence in their cytoplasm were considered positive, and the rest were considered negative (Fig. 2).

### Statistical analysis

Data were analyzed using the Kruskal-Wallis tests (Graph Pad InStat 3.1 software; San Diego, CA, USA) and *p*<0.05 was considered significant.

## RESULTS

### Experiment 1: Comparative analysis of sperm recovery after slow freezing and ultra-rapid freezing

Twenty-four normal semen samples were divided in half to compare sperm recovery after the application of two cryopreservation protocols: the currently accepted slow freezing procedure and the non-permeable cryoprotectant ultra-rapid freezing protocol. The semen parameters of the pre and post gradient processing "fresh" samples used to compare both cryopreservation protocols are listed in Table 1.

**Table 1:** Semen parameters of fresh samples prior to cryopreservation (mean ± SD).

Number of samples	24
Volume (ml)	3.2±1.2
Sperm concentration (10^6^/ml)	88.3±42.1
Progressive motility (%)	51.0±13.0
Non-progressive motility (%)	7.4±6.9
Immotile (%)	41.2±13.3
Post-gradient concentration (10^6^/ml)	75.2±34.6
Post-gradient progressive motility (%)	95.3±8.6

One to seven days after sperm cryopreservation the samples were removed from liquid Nitrogen and after thawing sperm concentration and motility were evaluated. No significant differences were found regarding sperm density. However, post ultra-rapid freezing samples exhibited significantly higher levels of progressive motility and lower levels of non-progressive and immotile sperm (Table 2).

**Table 2:** Comparative results after slow and ultra-rapid freezing techniques (mean ± SD).

Cryopreservation technique	Slow Freezing n: 24	Ultra-rapid freezing n: 24
Concentration (10^6^/ml)	39.0±19.9	38.8±11.9
Progressive motility (%)	16.6±7.4*	34.7±10.2**
Non-progressive motility (%)	9.0±4.0*	7.6±2.8**
Immotile (%)	74.4±10.1*	57.8±10.3**

(* , **) Significant differences (*p*<0.05)

### Experiment 2: Sperm DNA fragmentation comparison after slow and ultra-rapid freezing.

In experiment 2, a different set of 18 normal semen samples were employed to compare the cryopreservation protocols for their impact on DNA integrity. The semen parameters of the used samples are listed in Table 3. TUNEL assays were applied to the fresh post-gradient samples and to the final post-thaw samples that would be used for assisted reproduction, after the slow freezing and the ultra-rapid freezing procedures. The TUNEL values were significantly lower in the fresh post-gradient samples and in the post ultra-rapid freezing samples than in the post-slow freezing samples (9.1%, 14.6%, and 47.3%, respectively), exhibiting higher levels of DNA fragmentation after the slow freezing procedure (Table 4).

**Table 3:** Semen parameters analyzed prior to cryopreservation (mean ± SD).

Number of samples	18
Volume (ml)	3.1±1.2
Sperm concentration (10^6^/ml)	95.7±39.9
Progressive motility (%)	50.6±10.3
Non-progressive motility (%)	7.1±4.3
Immotile (%)	41.7±13.5
Post-gradient concentration (10^6^/ml)	85.3±48.9
Post-gradient progressive motility (%)	96.7±7.7
TUNEL value (%)	9.1±3.7

**Table 4:** Comparative TUNEL values for different cryopreservation techniques (mean ± SD).

Sample	Post-gradient separation	Post-slow freezing	Post-ultra-rapid freezing
TUNEL value (%)	9.1±3.7 *	47.3±13.4**	14.6±4.6*

(* , **) Significant differences (*p*<0.05)

**Figure 3:**
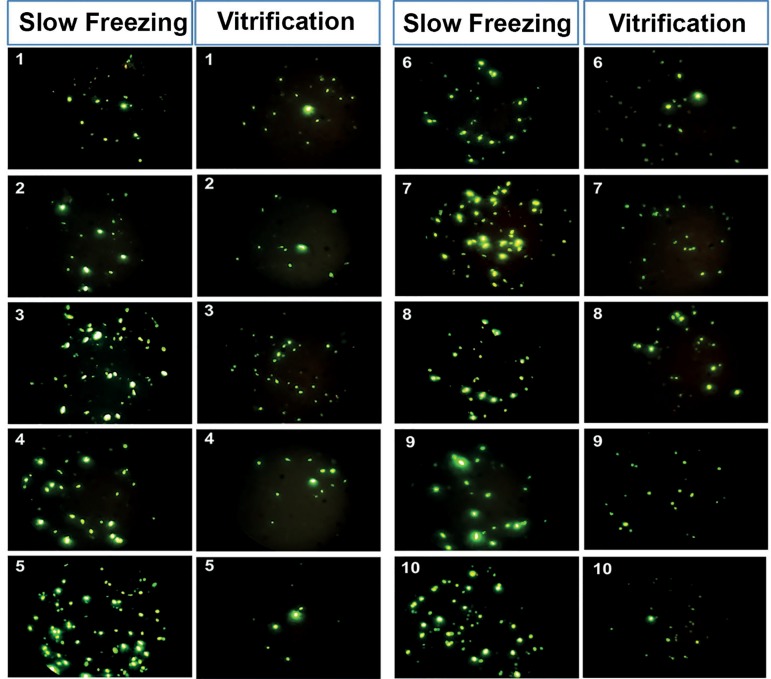
Comparison of spermatic DNA fragmentation by TUNEL-assay of the same 10 samples after slow freezing or ultra-rapid freezing (100X).

Figure 3 shows some examples of fluorescence obtained with the TUNEL assays of 10 samples after slow freezing and after ultra-rapid freezing.

## DISCUSSION

Gamete cryopreservation is a key procedure in ART. Many men look at sperm cryopreservation as a tool to preserve fertility and postpone fatherhood or as a means to save spermatozoa when they are diagnosed with severe male factor infertility or when they have to undergo chemotherapy, local radiotherapy or radical testicular surgery on account of malignant diseases. Sperm cryopreservation in different animal species dates from the 1950s, when slow freezing was established as the standard technique (Polge *et al*., 1949). Although slow freezing is at present the most extensively used technique in andrology laboratories for the cryopreservation of human spermatozoa, it has some drawbacks: the toxicity of cryoprotectants and the possible damages to sperm plasma membrane caused by ice crystallization during the cooling process. Moreover, it is a time-consuming and tedious procedure.

Vitrification, one of the initially developed cryopreservation techniques, has recently remerged (Luyet & Gehenio, 1940). The procedure is employed to cryopreserve cells using high concentrations of cryoprotectants and direct freezing in liquid nitrogen, allowing the suspension to form an ice crystal-free vitreous phase (Rall & Fahy, 1985). Avoiding ice crystal formation is the main advantage of this method, but it has to be performed carefully as cryoprotectants may be toxic at high concentrations. An additional benefit is the simplicity and short time required for its implementation. Vitrification has proven its efficacy from oocytes to blastocysts, completely replacing previously used slow freezing techniques. During the last decade, some studies addressing human sperm vitrification were published (Isachenko *et al*. 2004; Isachenko *et al*. 2004). Reports indicated low reproducibility until plastic containers were modified and sucrose was added to cryopreservation media to yield acceptable sperm recovery rates (Isachenko *et al*., 2008). The method applied in this study differed from conventional vitrification used for embryo and oocyte cryopreservation, since only sucrose, a non-permeable cryoprotectant, was employed; therefore, it was described herein as "ultra-rapid freezing".

Cryopreservation may induce high levels of apoptosis or DNA fragmentation in cells (Ortega Ferrusola *et al*., 2010; Kopeika *et al*., 2015). The aim of our study was to evaluate sperm DNA fragmentation levels after slow freezing and ultra-rapid freezing. The comparison of the TUNEL assay results showed that DNA damage was significantly higher in the samples processed via the slow freezing technique, suggesting that ultra-rapid freezing might be a safer alternative to preserve sperm DNA integrity. Our results confirmed the findings published by Isachenko *et al.* regarding the effectiveness of human sperm ultra-rapid freezing with non-permeable cryoprotectants (e.g.: sucrose) to preserve important semen physiological parameters such as progressive motility (Isachenko *et al.,* 2008). Our study revealed a comparative benefit offered by the ultra-rapid freezing technique regarding sperm DNA integrity that was not evident in previous reports (Isachenko *et al*. 2004; Isachenko *et al*. 2004).

Motility is related, among other factors, to sperm DNA integrity (Ngamwuttiwong & Kunathikom, 2007; Yildiz *et al*., 2007). Our study found higher post thaw motility in the ultra rapid freezing method than in the slow freezing protocol, possibly because DNA from vitrified sperm experienced less damage when exposed to ultra-rapid freezing and low concentrations of non-permeable cryoprotectants. In coincidence with the DNA integrity results evaluated by the TUNEL technique, higher levels of DNA fragmentation and lower sperm motility after thawing were observed when the conventional slow freezing technique was applied. Other factors affecting sperm motility after freezing include changes in the plasma membrane, mitochondrial damage, and increased production of reactive oxygen species (Desrosiers *et al*., 2006; Yildiz *et al*., 2007), but none of these factors were analyzed in this study.

The present findings may have an impact on ART results, as it is widely known that damaged sperm DNA may result in poor quality embryos (Benchaib *et al*., 2003). In our study using normal semen samples, sperm motility after ultra-rapid freezing was significantly higher than after slow freezing. This may allow, in some cases, the use of conventional IVF, avoiding the intracytoplasmic sperm injection (ICSI) procedure and its known limitations when compared to in vitro fertilization (IVF) (Dumoulin *et al*., 2000; Shalom-Paz *et al*., 2011).

If future studies employing pathologic semen samples confirm the present findings, the use of ultra-rapid freezing may be extended to testicular biopsy specimens, since embryos produced from cryopreserved testicular sperm tend to exhibit higher fragmentation rates with poorer embryo quality and lower implantation and pregnancy rates (Benchaib *et al*., 2007). Post-thaw increased motility and better protection of DNA integrity provided by the ultra-rapid freezing technique described previously might have an impact on the results of ART procedures with testicular sperm retrieval. A prospective study on the issue has been started in our IVF unit.

Our results, although promising, are limited by the following factors: the small size of our sample; the fact that they were all normal samples from young donors; and by the limitations of the TUNEL technique to assess DNA integrity. A prospective randomized multicenter trial is required before this promising procedure is put to use in clinical settings.

## CONCLUSION

The present data indicated that ultra-rapid freezing of human sperm with non-permeable cryoprotectants, referred to herein as ultra-rapid freezing, might be a more effective and safer alternative to the slow freezing technique currently in use.
